# Toward an understanding of when prior knowledge helps or hinders learning

**DOI:** 10.1038/s41539-021-00103-w

**Published:** 2021-08-19

**Authors:** Garvin Brod

**Affiliations:** 1 DIPF|Leibniz Institute for Research and Information in Education, Frankfurt am Main, Germany; 2 Goethe University, Frankfurt am Main, Germany

**Keywords:** Human behaviour, Human behaviour

Knowledge begets knowledge—or so they say? David Ausubel^1^ has famously described prior domain knowledge as the most important determinant of a student’s learning success, “ascertain this and teach him accordingly”. Indeed, prior knowledge—previously learned information organized in a learner’s memory^2^—has long been known to explain large portions of variance in learning outcomes^3^. A new meta-analysis by Simonsmeier et al.^4^ suggests that this is not the end of the story, however. By distinguishing learning outcomes from learning gains, the new meta-analysis found that prior knowledge indeed explained large portions of variance in learning outcomes, but it did not—on average—explain variance in learning gains. The former result indicates that those students in a class who know the most at the beginning of a class will likely know the most at the end as well. The latter result is an eye-catching one, as it suggests that knowledge at the beginning of a class does not, in fact, determine how much a student will learn from a particular task or instruction. Does this finding contradict the famous statement by Ausubel? At the very least, as put by Simonsmeier et al., it “calls for systematic research on the conditions under which prior knowledge has positive, negative, or negligible effects on learning”^4^. I wholeheartedly agree that this is of outmost importance for both theory construction and educational practice, and I applaud the authors of the meta-analysis for bringing this topic to the fore. The goal of this brief commentary is to provide initial pointers for systematic research on the factors that determine whether and how prior knowledge affects learning.

Whether and how prior knowledge exerts an influence on learning certainly depends on the prior knowledge itself. The different dimensions of prior knowledge and their effect on learners’ text comprehension have been thoroughly described in a recent article^5^, which identified four important dimensions: amount, accuracy, specificity, and coherence (for earlier work on mapping and defining prior knowledge, see refs. ^6,7^). The existence of different dimensions of prior knowledge makes it clear that the effect that prior knowledge has on learning depends on more than just having more or less knowledge available. As will be further explained below, however, looking only at the knowledge itself is not sufficient, as even large amounts of correct, specific, and coherent prior knowledge can be unused.

In this short commentary, I will argue that there are several determinants for whether and how prior knowledge affects learning. Put differently, the identical prior knowledge can steer learning differently in different learning tasks and can thus both help and hinder learning. I will focus on three determinants: (1) whether prior knowledge is activated (i.e., information is retrieved from memory), (2) whether it is relevant for the learning task at hand, and (3) whether it is congruent or incongruent with the to-be-learned content. These three determinants can be put in a hierarchical relation (see Fig. 1), which illustrates that, for example, relevance of the prior knowledge only becomes important when this knowledge is activated. Note that this means that the determinants considered here are all acting on an intra-individual level. Inter-individual and environmental determinants such as participants’ age or the duration of the intervention, which are covered in the meta-analysis as well, are not considered. While the different determinants are presented as dichotomies, these dichotomies are meant as the extreme ends of one scale/dimension. There is substantial evidence that each of the dimensions has a strong impact on learning, which I will elaborate on in the following.

**Fig. 1:**
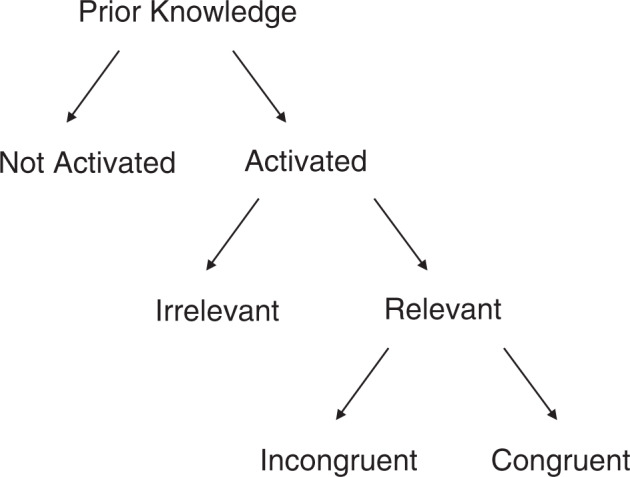
Determinants for whether and how prior knowledge affects learning. Note that, while the different determinants are presented as dichotomies, these dichotomies are meant as the extreme ends of one scale/dimension.

## Determinants of the effects of prior knowledge on learning

To have any effect on learning whatsoever, prior knowledge needs to be activated first. That is not trivial, as has been illustrated in a seminal study by Bransford and Johnson^8^. Participants had to read a description of an activity of which all participants could be assumed to have substantial prior knowledge (i.e., washing clothes). Some participants received a hint (i.e., the topic of the passage) beforehand that enabled the activation of relevant prior knowledge, whereas other received the hint afterward or not at all. Comprehension ratings and recall performance were strongly enhanced in participants who were given the hint beforehand, which provides evidence that available prior knowledge needs to be activated in order to affect learning. The phenomenon that prior knowledge is not activated by learners has been particularly well researched in children, who display characteristic deficiencies in using their prior knowledge strategically, which hampers their learning performance^9–11^. In summary, it is not sufficient that prior knowledge is available but it also has to be activated and used to steer the learning process.

Even if some prior knowledge gets activated by learners, it has to be relevant for the learning task at hand to have a beneficial effect. Research on the so-called “Baker–baker paradox”^12^ illustrates the importance of this dimension. The paradox describes the finding that remembering a face–name association (i.e., that a person’s surname is Baker) is disproportionately harder than remembering face–profession associations (i.e., that a person’s profession is baker). In the case of face–name associations, even if participants knew someone with the surname Baker and activated this prior knowledge, they had a hard time to leverage it to connect this surname to a new face in a meaningful way because of the arbitrariness of the association^13^. Their activated prior knowledge is, thus, largely irrelevant for the learning task at hand. In contrast, in the case of profession–name associations, prior knowledge is relevant because a common profession activates a large knowledge network that participants reported to use to associate the profession with the face (e.g., by imagining the face with a baker’s hat or by evaluating facial characteristics as to whether these fit with their idea of a baker)^13^. In summary, research on the “Baker–baker paradox” shows that knowledge can be more or less relevant in a particular learning context.

Activated irrelevant prior knowledge can even hamper learning. Research on memory intrusions suggests that large amounts of correct prior knowledge in a domain can have detrimental consequences for learning in this domain because it can induce perceptual biases and intrusions. For example, football experts who studied lists of animal names that were also names of football teams later falsely recalled many non-presented animal names that represented football teams. This resulted in a higher number of falsely recalled words in experts than in non-experts, a pattern that was absent in a control task in which participants had to study body parts^14^. Higher (activated) prior knowledge can thus lead astray when it is irrelevant for the learning task at hand.

Even if prior knowledge is activated and relevant, it has to be congruent (i.e., in agreement) with the to-be-learned information to have an unequivocal beneficial effect. Congruency describes the fit or agreement between prior knowledge and new information. A wealth of memory research has demonstrated that new information that is congruent with prior knowledge tends to be better remembered than information this is incongruent with it; a phenomenon that has been dubbed the *memory congruency effect* and that has been suggested to arise from a more elaborate memory trace that is laid down during encoding and facilitated memory search during retrieval^15–17^. While the memory congruency effect suggests that higher congruency between prior knowledge and new information typically goes along with better learning of the new information, the opposite can for once be true as well. Highly incongruent new information has been shown to be learned well, but only when it leads to strong surprise in learners^18^.

Research on conceptual change has dealt extensively with different types or degrees of (in)congruency that exist between prior knowledge and new information and the way that this affects whether prior knowledge is helping or hindering learning. These differences range between what has been called the *enrichment* kind and the *radical conceptual change* kind^19^, which point to differences in the kind of change in the learner’s knowledge structures that is necessary to incorporate the to-be-learned information. These differences can be illustrated by research on the acquisition of taxonomic knowledge^20^. Consider a taxonomy of the concept “whale”. Learning (a) that whales can be found in the Arctic Ocean, (b) that orcas (killer whales) belong to the family of dolphins, and (c) that whales are mammals are all likely to be new to most learners. However, these three arguably differ in the kind of change in the learner’s taxonomy that is necessary to incorporate the to-be-learned information^20^: (a) can be fairly easily integrated because it does not require larger reorganization (i.e., enrichment of the existing taxonomy); (b) requires differentiation, which goes along with a reappraisal of which attributes of a concept are primary and which are secondary; (c) requires large-scale reorganization of the hierarchy, that is, shifting of a concept from one branch to another in the taxonomy. To conclude, the greater the necessary reorganization of the prior knowledge, the more difficult it will be for learners to acquire new information.

## The complex relation between prior knowledge and learning success

The goal of this commentary is to provide initial pointers for systematic research on the factors that determine whether and how prior knowledge affects learning. I have suggested that there are at least three important determinants that need to be taken into account besides the structure of the prior knowledge itself: whether prior knowledge is activated, whether it is relevant, and whether it is congruent with the to-be-learned information. While there is no simple one-to-one mapping between those determinants and learning success, they do provide a glimpse into the complexity of the relation between prior knowledge and learning success. Figure 1 illustrates that there are many contexts in which prior knowledge will likely not be beneficial for learning. Only when it is activated, relevant, and congruent will prior knowledge reliably help. When it is not activated at all, the prior knowledge a learner brings to the learning task will have negligible effects on learning outcomes. When the prior knowledge that is activated is irrelevant for the task at hand, its effects will either be negligible or they will even hinder learning because of intrusions or biases. Prior knowledge that is activated, relevant, and incongruent with the to-be-learned information will mostly hinder learning, too (see, e.g.^21^).

Figure 1 also illustrates why the overall effects reported in the meta-analyses by Simonsmeier et al.^4^, which suggest a wide distribution of effect sizes that centers around zero, might not be that surprising after all. While the meta-analysis covered an impressive number of inter-individual and environmental moderators of the effect of prior knowledge on learning gains, it did not explicitly consider the determinants described in the current article except for the knowledge itself. While it can be assumed that most of the knowledge that was assessed during pretests was relevant for the learning task at hand, it is unclear whether this knowledge was activated by the learners during the task and how congruent it was with the to-be-learned content. Of note, the meta-analysis did include studies that have targeted incongruent prior knowledge by measuring the amount of incorrect knowledge regarding the to-be-learned concept (i.e., misconceptions). None of these studies reported the correlation with learning gains, however, which impeded further analyses.

Coming back to the initial question: does the famous statement by Ausubel^1^, according to which prior knowledge is the most important determinant of a student’s learning success, need to reconsidered given the findings of the meta-analysis? Not so fast. First of all, one has to say that he did *not* say that prior knowledge will always be helpful, but only that it will be a strong determinant of what a student will learn in a lesson. In a similar vein, Simonsmeier and colleagues^4^ suggest that the different ways in which prior knowledge affects learning, while all important, might sometimes cancel each other out. I will thus conclude by saying that it remains of outmost importance to assess a learner’s prior knowledge before teaching her some new content. Unraveling the systematics of whether and how this prior knowledge then steers the learning process is food for future research.

## Data Availability

There are no data attached to this manuscript.

## References

[1] Ausubel, D. P. *Educational Psychology: A Cognitive View* (Holt, Rinehart and Winston of Canada Ltd, 1968).

[2] Shing YL, Brod G (2016). Effects of prior knowledge on memory: implications for education. Mind Brain Educ..

[3] Dochy F, Segers M, Buehl MM (1999). The relation between assessment practices and outcomes of studies: the case of research on prior knowledge. Rev. Educ. Res..

[4] Simonsmeier, B. A., Flaig, M., Deiglmayr, A., Schalk, L. & Schneider, M. Domain-speficic prior knowledge and learning: a meta-analysis. *Educ. Psychol*. 2021, in press. 10.1080/00461520.2021.1939700

[5] Mccarthy KS, Mcnamara DS (2021). The multidimensional knowledge in text comprehension framework. Educ. Psychol.

[6] Dochy FJRC, Alexander PA (1995). Mapping prior knowledge: a framework for discussion among researchers. Eur. J. Psychol. Educ..

[7] Alexander PA, Schallert DL, Hare VC (1991). Coming to terms: how researchers in learning and literacy talk about knowledge. Rev. Educ. Res..

[8] Bransford JD, Johnson MK (1972). Contextual prerequisites for understanding: Some investigations of comprehension and recall. J. Verbal Learn. Verbal Behav..

[9] Barnes MA, Dennis M, Haefele-Kalvaitis J (1996). The effects of knowledge availability and knowledge accessibility on coherence and elaborative inferencing in children from six to fifteen years of age. J. Exp. Child Psychol..

[10] Bjorklund DF, Miller PH, Coyle TR, Slawinski JL (1997). Instructing children to use memory strategies: evidence of utilization deficiencies in memory training studies. Dev. Rev..

[11] Schneider W, Sodian B (1997). Memory strategy development: lessons from longitudinal research. Dev. Rev..

[12] McWeeny KH, Young AW, Hay DC, Ellis AW (1987). Putting names to faces. Br. J. Psychol..

[13] Cohen G (1990). Why is it difficult to put names to faces?. Br. J. Psychol..

[14] Castel AD, McCabe DP, Roediger HL, Heitman JL (2007). The dark side of expertise: domain-specific memory errors. Psychol. Sci..

[15] Brod G, Shing YL (2019). A Boon and a Bane: comparing the effects of prior knowledge on memory across the lifespan. Dev. Psychol..

[16] Schulman AI (1974). Memory for words recently classified. Mem. Cogn..

[17] Bein O (2015). Delineating the effect of semantic congruency on episodic memory: The role of integration and relatedness. PLoS ONE.

[18] Brod G, Hasselhorn M, Bunge SA (2018). When generating a prediction boosts learning: the element of surprise. Learn. Instr..

[19] Chi, M. T. H. Three Types of Conceptual Change: Belief Revision, Mental Model Transformation, and Categorical Shift. in *Handbook of research on conceptual change* (ed. Vosniadou, S.) 61–82 (Erlbaum, 2008).

[20] Thagard P (1990). Concepts and conceptual change. Synthese.

[21] Alvermann DE, Smith LC, Readence JE (1985). Prior knowledge activation and the comprehension of compatible and incompatible text. Read. Res. Q..

